# Opportunities and Challenges to Microbial Symbiosis Research in the Microbiome Era

**DOI:** 10.3389/fmicb.2020.01150

**Published:** 2020-06-16

**Authors:** Suhelen Egan, Takema Fukatsu, M. Pilar Francino

**Affiliations:** ^1^Centre for Marine Science and Innovation (CMSI), School of Biological, Earth and Environmental Sciences (BEES), UNSW Sydney, Sydney, NSW, Australia; ^2^Bioproduction Research Institute, National Institute of Advanced Industrial Science and Technology (AIST), Tsukuba, Japan; ^3^Joint Research Unit in Genomics and Health, Fundació per al Foment de la Investigació Sanitária i Biomèdica de la Comunitat Valenciana (FISABIO)/Institut de Biologia Integrativa de Sistemes (Universitat de València i Consejo Superior de Investigaciones Científicas), València, Spain; ^4^CIBER en Epidemiología y Salud Pública, Madrid, Spain

**Keywords:** microbiome, symbiosis, microbial interactions, microbiota (microorganism), host-microbe association

The large extent to which microorganisms have influenced the evolution of life on our planet is unquestionable. These unseen players have been appreciated for their role in health and disease since the late 19th century, however it is only relatively recently that the breadth of these interactions has been revealed. For example, we now know humans are hosts to trillions of microbes some of which can impact our physiology, development, nutrition, health and even influence our behavior (Knight et al., 2017; Cani, 2018; Francino, 2018; Johnson and Foster, 2018). Syntrophic (cross-feeding) interactions between diverse soil microbes enhance the rate of nutrient cycling, providing essential ecosystem services and stimulating plant growth (Jansson and Hofmockel, 2018; Dubey et al., 2019). Knowledge of the partnership between invertebrates and sulfur-oxidizing bacteria has facilitated our understanding of how entire ecosystems flourish in the deep sea (Dubilier et al., 2008) and there is now unquestionable support for the endosymbiotic origin for eukaryotic organelles (Archibald, 2015). Indeed, as knowledge of the microbial world increases, we can confidently argue that all living organisms form some type of symbiotic relationship with microorganisms that influence their evolutionary success.

The purpose of this Grand Challenge article is to provide our personal perspective on the current state of microbial symbioses research and provide a reflection on the main challenges and opportunities this field faces moving forward. While we make reference to specific symbiotic systems this article is not intended to be an exhaustive review of microbial symbioses and the reader is encouraged to go to some excellent reviews on this topic for more information [e.g., Morris et al. (2013); Douglas (2015); Knight et al. (2017); Sanchez-Canizares et al. (2017); D'Souza et al. (2018); Lynch and Hsiao (2019); Rodriguez et al. (2019); Wilkins et al. (2019); Lemoine et al. (2020); Teichman et al. (2020)].

The term symbiosis is often used to describe mutually beneficial interactions between two organisms, however the meaning of the term in Greek is “living-together” and it was initially coined by the German botanist Professor Heinrich de Bary in 1878 to describe any relationship between two different organisms in which at least one benefits [see Egerton (2015); Oulhen et al. (2016)]. As such we define microbial symbioses as interactions involving at least one microbial partner where either all partners benefit (cooperation or mutualism); one benefits and the other/s is harmed (parasitism or pathogenesis); one (or many) benefits and the other/s is neither harmed nor benefited (commensalism) (Figure 1). Although these definitions are key for communication it is important to recognize that symbioses rarely fall strictly into one of these categories. Relationships are often fluid and can be influenced by a variety of factors including genetics, age and environmental conditions, therefore it is paramount to view symbioses as a gradient rather than in absolute terms. In this context even near-zero benefit relationships should be considered under the term symbioses.

**Figure 1:**
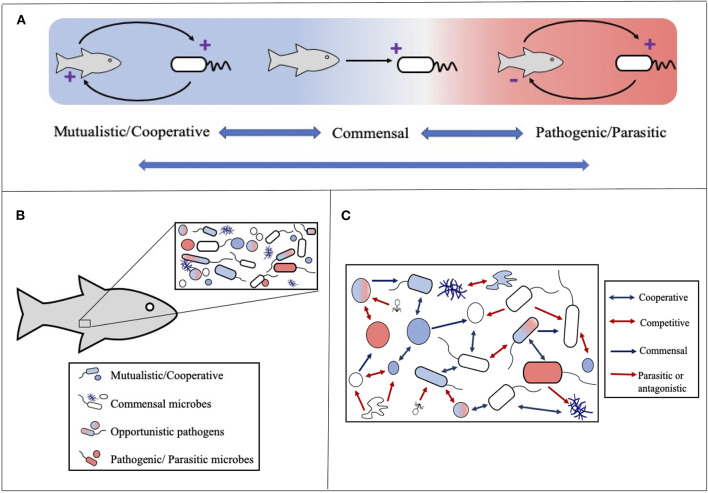
The main types of microbial symbioses. **(A)** Microbial interactions range from mutually beneficial to harmful for one or more partners. Blue double headed arrows highlight that relationships can move between classifications often influenced by environmental conditions. **(B)** Host-microbe symbioses should be considered within the context of microbial communities where the host participates in multiple and often different symbiotic relationships. **(C)** Microbial communities are influenced by a variety of microbe-microbe symbioses ranging from cooperation (e.g., syntrophy or co-metabolism) to competition. Arrows depict generally beneficial (blue) and detrimental (red) outcomes for one (single arrowhead) or both (double arrowhead) members. Note as with host-microbe symbioses these relationships can be viewed as fluid and influenced by environmental conditions.

The past two decades have seen an increase in research related to microbial symbioses with research publications increasing from ~640 per year in 1998 to ~9,350 per year in 2018, representing a proportional increase among microbiology related publications from 4.6 to 12.8% (Figure 2). This increased interest could be due to a number of advancements in the field of microbial symbioses, arguably the most impactful of which has been the “microbiome boom”. The advent of next generation sequencing technologies (NGS) heralded the development of high throughput tools that could characterize microbial communities at unprecedented speed and depth. Because many microbial symbionts are yet to be cultured, culture independent methods such as amplicon sequencing and shotgun metagenomics allowed biologists to get some of the first glimpses of symbiont diversity and function. The significance of these technological advances might be best exemplified by the human microbiome project (Lloyd-Price et al., 2017). This project and many other studies worldwide have provided an understanding of microbial diversity and function in the human microbiome well beyond what had been attained through culture-based analyses. Not only did this influence the way we see ourselves as individuals, but the associated publicity put a spotlight on microbial symbioses and researchers came to appreciate the microbiome associated with their system of interest. Awareness of the important function of the microbiome in the development and health of an organism has resulted in many biologists to consider their host system and the associated symbionts as a “holobiont” or “meta-organism” (Egan et al., 2013; Rosenberg and Zilber-Rosenberg, 2016; Webster and Thomas, 2016; Sanchez-Canizares et al., 2017; van de Guchte et al., 2018). However, the holobiont and by extension the hologenome concept remain controversial (Moran and Sloan, 2015; Douglas and Werren, 2016) particularly as they pertain to the host and its microbiome as a single evolutionary unit. In order to validate the holobiont concept from an evolutionary perspective, new theoretical approaches are needed that acknowledge the different levels at which natural selection can operate in the context of microbiome-host interactions. For example, selection could occur at the level of the holobiont when a transgenerational association among specific host and symbiont genotypes can be maintained. Continued research and interdisciplinary collaboration on this topic will greatly improve our understanding of the holobiont concept and its place in the ecology and evolution of plants and animals. Nevertheless, the holobiont concept has resulted in a shift from the focus on symbioses involving one microbial partner and a single host (e.g., squids and luminescent *Allivibrio*; legumes and *Rhizobium*; aphids and *Buchnera*) and toward a greater interest in symbioses comprising complex multi-partner consortia (e.g., animal gut systems, marine invertebrates, plant and seaweed epiphytes, microbe-microbe interactions in soil or aquatic biomes etc.). Moreover, there is a realization that even the relatively well understood binary symbioses such as aphids and *Buchnera* are more complex with a number of diverse facultative symbionts contributing to resistance to parasites (Oliver et al., 2003), expanding host plant usage (Tsuchida et al., 2004) and temperature adaptation (Montllor et al., 2002).

**Figure 2:**
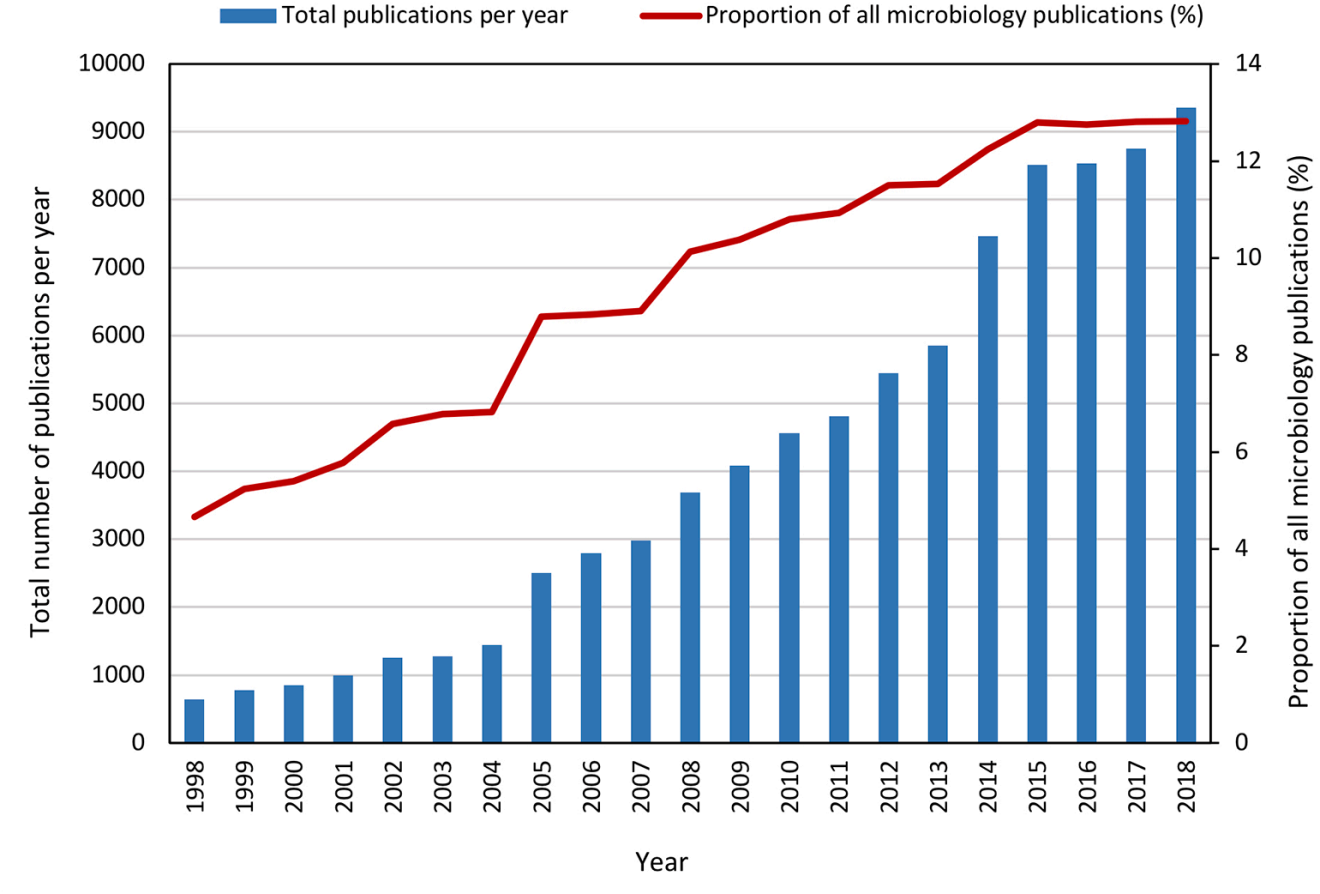
Number of publications (review or original article only) 1998–2018 retrieved from Scopus database search (November 2019) with keyword search Microb* AND Symbio* or Microb* AND Host*.

Despite our increased knowledge of the diversity of microbial symbionts, our understanding of the functional role and mechanisms of host interaction of these symbionts is still limited. However, combining -omic information with advanced imaging techniques (e.g., Nanoscale secondary ion mass spectrometry (NanoSIMS) imaging, bioorthogonal noncanonical amino acid tagging (BONCAT), confocal Raman microscopy, fluorescence *in situ* hybridization (FISH), atomic force microscopy) is helping to fill this knowledge gap. For example, using NanoSIMS imaging, Tarquinio and colleagues (Tarquinio et al., 2018) recently demonstrated that leaf-associated microbiota is responsible for facilitating dissolved organic nitrogen uptake in seagrasses thus enhancing growth of this important habitat-forming species.

Questions about symbiont acquisition and the stability of host-microbe relationships also remain. These questions are particularly pertinent given that we live in a changing climate and there are now a number of publications reporting on microbiome adaptation to the Anthropocene (Putnam et al., 2017; Pita et al., 2018; Amato et al., 2019; Cheng et al., 2019). Microbial dysbiosis (or disequilibrium of the microbial community) resulting in disease is a common consequence of environmental stress on holobionts, with syndromes such as inflammatory bowel disease (IBD), psoriasis, coral disease and algal bleaching being attributed to a microbial imbalance and/or rise of microbial opportunists (West et al., 2015; Egan and Gardiner, 2016). Therefore, identifying the tipping points where symbiotic interactions fail will be an important area of future research. Paradoxically, flexible symbioses may also be key to the ability of a host organism to adapt to environmental change. Manipulation of microbial symbionts has been successfully applied to both animal and plant systems (Goodman et al., 2011; Crotti et al., 2012; Vorholt et al., 2017; Bober et al., 2018; Brugman et al., 2018). Building on these advances recent studies have also provided proof of concept for microbiota manipulation (Damjanovic et al., 2019) and the potential health benefits for coral reefs (Rosado et al., 2019). Given the increasing pressure on both engineered and natural ecosystems, selection of resilient microbiota and development of synthetic symbioses are expected to be growth areas for microbial research [e.g., see Mueller and Sachs (2015); Peixoto et al. (2017); Herrera Paredes et al. (2018); Vrancken et al. (2019)]. The results of this work could help to protect vulnerable habitats and our health from the consequences of a rapidly changing climate.

In order to address the current knowledge gaps in microbial symbioses the field must overcome some conceptual and technical challenges, including the reliance on correlative data to explain causation and the ability to disentangle the importance of individuals in complex symbiosis systems. The availability of genetic tools and of obtaining axenic or gnotobiotic hosts is a key step in the move from a reliance on correlative data toward hypothesis testing and establishing causation. These tools open the door to explore functional roles, mechanisms of interactions and potential redundancies across and within different symbiont systems. However, genetic systems and microbe-free (or reduced) hosts are only readily available for a handful of well-studied laboratory-based model organisms (e.g., mouse, zebrafish, *Caenorhabditis elegans, Drosophila, Arabidopsis, Hydra*). While these models continue to advance our knowledge of symbiosis, see Bosch et al. (2019), they do not fully capture the diversity and complexity of the majority of natural systems. Thus, the development of axenic cultures and genetic manipulation tools for a diverse range of symbiotic systems represent opportunities for future research that will help to address many of the current challenges facing the field of microbial symbioses. As many symbionts supply important growth factors or perform key metabolic functions for the host, obtaining axenic cultures is a challenge. However current advancements in metabolic pathway reconstruction and network modeling (Bosi et al., 2017; Pan and Reed, 2018; Thommes et al., 2019) provide an opportunity to identify metabolic exchanges and/or growth requirements (Jijakli and Jensen, 2019; Burgunter-Delamare et al., 2020) that may assist in the rational design of culturing methods for both symbionts and axenic hosts. Such methods have successfully been used to cultivate fastidious pathogens (Renesto et al., 2003) and members of the human microbiota (Lagier et al., 2018). Development of these tools will require a “renaissance” in cultivation and classical molecular biology and it is possible that one of the greatest challenges will be to convince young scientists to invest in these areas of research despite the current “big data” research culture.

This is an exciting time for the field of microbial symbioses. The past decade has generated a wealth of information highlighting the diversity of microbial interactions. Together with a number of important technology breakthroughs (e.g., CRISPR-Cas, nanoscale imaging, single cell genomics) we are now in a position to move beyond the exploratory phase of microbial symbioses research. The field is also benefiting from an open dialog between researchers from diverse disciplinary backgrounds and/or working on distantly related symbiotic systems. These scientific collaborations will be important to identify common traits and obtain a holistic understanding of diverse microbial symbioses. Given the extent to which microbial symbioses are likely to impact an organism's wellbeing, with a greater understanding of the mechanisms, ecology and evolution of microbial symbioses, we will be better positioned to address the 21st century economic, environmental and human health challenges.

## Author Contributions

SE wrote the first draft of the manuscript. TF and MF contributed to the writing and approved the final version of the manuscript.

## Conflict of Interest

The authors declare that the research was conducted in the absence of any commercial or financial relationships that could be construed as a potential conflict of interest.
